# Efficiency of Galanti and Guisti Method of ADA Estimation in Comparison with the Gold Standard

**DOI:** 10.4314/ejhs.v30i6.7

**Published:** 2020-11

**Authors:** Manasa Reddy Dubba, V Ramyashree, SS Harshitha, Monalisa Biswas, Revathi P Shenoy, Varashree Bolar Suryakanth

**Affiliations:** Kasturba Medical College, Manipal, Manipal Academy of Higher Education, Manipal, Karnataka, India; 2 Department of Biochemistry, Kasturba Medical College, Manipal, Manipal Academy of Higher Education, Manipal, Karnataka, India; 3 Department of Biochemistry, Kasturba Medical College, Manipal, Manipal Academy of Higher Education, Manipal, Karnataka, India; 4 Department of Biochemistry, Kasturba Medical College, Manipal, Manipal Academy of Higher Education, Manipal, Karnataka, India-576104; 5 Department of Biochemistry, Kasturba Medical College, Manipal, Manipal Academy of Higher Education, Manipal, Karnataka, India-576104; 6 Department of Biochemistry, Kasturba Medical College, Manipal, Manipal Academy of Higher Education, Manipal, Karnataka, India-576104

**Keywords:** Adenosine Deaminase, Galanti and Guisti method, TB, SCID

## Abstract

**Background:**

Adenosine Deaminase, the key enzyme of purine metabolism catalyzing the irreversible hydrolytic deamination of adenosine to inosine is implicated in a varied spectrum of human diseases ranging from SCID to TB and pneumonia. Estimation of ADA offers an easy, relatively affordable and reliable diagnostic alternative and/ or adjunct (specially in a TB endemic nation) which emphasizes the necessity of a feasible and implementable alternative method to the Diazyme method of ADA estimation requiring high end autoanalyzer and infrastructural setup.

**Methods:**

Sixty body fluids samples (irrespective of gender, age, diagnosis or sample type) received by the Clinical Biochemistry Laboratory, Kasturba Medical College, Manipal for fluid ADA estimation by the Diazyme assay method (cobas 6000) was simultaneously processed by the Galanti and Guisti manual method to estimate the comparability and the aggregability of results obtained by the two analytical techniques.

**Results:**

The Galanti and Guisti manual method of ADA estimation showed aggregability with the Diazyme autoanalyzer method for 90% of the assayed study samples with the manual method uniformly showing higher values when compared to the analyzer method. A correction factor of 2.44 was arrived at which could effectively achieve comparability between the two assay methods.

**Conclusion:**

The Galanti and Guisti manual method of ADA estimation might be a feasible, rapid, reliable and cost-effective method for estimation of fluid ADA when compared to the cost and infrastructure intensive autoanalyzer.

## Introduction

Adenosine Deaminase (Adenosine aminohydrolase EC 3.5.4.4) is an enzyme of purine metabolism which catalyses the irreversible hydrolytic deamination of adenosine to inosine and ammonia (1). ADA is the key enzyme of purine metabolism with a high degree of conserved amino acid sequence which suggests the essential and indispensable role of ADA in the purine salvage pathway (2). ADA is a ubiquitously expressed enzyme with its major activity localized in the cytosolic compartment (only 3% in subcellular organelles) and the highest activities recorded in the lymphoid tissues, particularly the thymus, the brain, spleen and the gastrointestinal tract (1,3).

ADA is involved in the development and maintenance of the immune system as it ensures the breakdown of metabolic by-products toxic to T-lymphocytes, in absence of which the toxic by-products of purine metabolism kill the T cells shortly after they are produced in the bone marrow, greatly reducing the number of T cells and resulting in ADA dependent severe acute combined immunodeficiency (SCID) (3). Whilst most notable affects are on lymphocytes, given the ubiquitous nature of the enzyme, several non-immunological manifestations including neurodevelopmental deficits, pulmonary manifestations associated with pulmonary-alveolar proteinosis, sensorineural deafness and skeletal abnormalities are observed (3). ADA has also been associated with epithelial cell differentiation, neurotransmission, maintenance of gestation, stimulation of the release of excitatory amino acids and the coupling of A1 adenosine receptors and heterotrimeric G proteins (2,4). While elevated levels of ADA have been associated with typhoid fever, infectious mononucleosis, tuberculous meningitis (differentiates tuberculous from viral lymphocytic meningitis), AIDS, rheumatoid arthritis, psoriasis, pneumonia, chronic diarrhoea and widespread skin rashes (5). Assessment of ADA in pathologic fluids has been found to be of immense value in the diagnosis of tuberculosis, a common communicable condition affecting a significant number of individuals across the globe (6). Estimation of pleural fluid ADA could be easy, cheap diagnostic alternative offering high sensitivity and specificity test for diagnosis of TB pleural effusion and circumventing the need for pleural biopsy to ascertain the diagnosis of TB about 40% of the patient population (7,8).

Given the wide range of functions, the biochemical estimation and quantification of ADA is a diagnostic and prognostic aid to a myriad clinical condition. However, estimation of ADA remains a cost-intensive infrastructural hurdle in resource constrained rural predominant settings of India, where approximately 2.69 million TB cases and 9700 deaths associated with TB are reported annually, the country being the leading contributor to the global burden of TB (9,10). According to the World Health Organisation, 30 countries accounted for 87% of new TB cases in the year 2018, with eight countries accounting for two thirds of the global burden and India being the leading contributor of the global burden (10). Thus, it is clear that tuberculosis still remains a public health hazard in India with an estimated 40% of the Indian population being infected with TB bacteria; the vast majority of whom have latent TB rather than TB disease (9,11). However, with rapid diagnosis and interventions, TB is indeed curable and preventable too. Identification of TB in body fluid still remains a challenging area in diagnostics with fluid ADA activity offering a welcome diagnostic alternative and adjunct for diagnosis of TB, estimation of ADA being comparatively inexpensive, non-invasive, less time consuming and reliable (12). Literature evidences have proved its utility of ADA in the diagnosis of TB with the hypothesis that the observed increase in ADA activity is possibly due to delayed hypersensitivity reaction to mycobacterial antigen (12–14). ADA with its relative ease of sampling requirement, assay and speed of result availability (on the same day compared 2 weeks required for culture) indeed offers an immense potential as an adjunct diagnostic tool for TB (associated forms and conditions) and other ADA related pathologies like SCID, allowing rapid institution of treatment leading to reduction in complication and improvement in patient outcome (15). This dictates the imperative need of a feasible and implementable alternative to the diazyme (kit) method (using 6000 autoanalyzer platform) of ADA estimation which could help in fast tracking the diagnosis of ADA implicated diseases and also ensure cost management in terms of reduced need of sample outsourcing and testing services to distant laboratories. Thus, we aim to evaluate the diagnostic efficiency of the cost-effective age old Galanti and Guisti manual method of ADA estimation and compare the accuracy of this method with the cost intensive, high end infrastructure requiring autoanalyzer assayed kit method.

## Methods

The study was approved by the Institutional Ethical Committee and carried out in the Department of Biochemistry for a period of six months. Sixty samples of body fluids received by the Clinical Biochemistry Laboratory (physician requested ADA estimation) was included in the study; no additional body fluid samples were collected. All samples received for ADA estimation were included in the study regardless of gender, age, diagnosis or the type of sample (CSF, pleural, ascitic). As per the standard operating protocol of the laboratory, all the fluid samples were analysed by the routine diazyme ADA assay by cobas 6000 (Roche diagnostics). Residual fluid samples were used for ADA estimation by the manual Galanati and Guisti method to assess the comparability between the two methods. Further, relevant haematological and biochemical laboratory findings were retrieved through the Laboratory Information System. Clinical history, and diagnostic details of the patients were obtained from patient records stored in the Medical Records Department of the hospital. Confidentiality of patient was respected and uncompromised during the entire study.

**Statistical Analysis**: Mean difference, bias, Standard Deviation and the upper and lower limits of agreement were determined. A Band Altman plot was generated to estimate the degree of agreement between the two analytical methods. Linear regression analysis was carried out to generate the regression equation for the two assay methods.

## Results

The ADA values obtained from both the assay method (analyser and manual method) were recorded. It was observed that the manual Galanti and Guisti method of ADA estimation uniformly showed higher values for ADA when compared to the values generated by the analyser method. We arrived at a correction factor of 2.44 to achieve comparability between the two assay methods. Therefore, diving the results obtained from Galanti and Guisti method by a factor of 2.44, could approximately provide a value similar to that obtained from the Diazyme autoanalyzer assay method.

**Figure 1:**
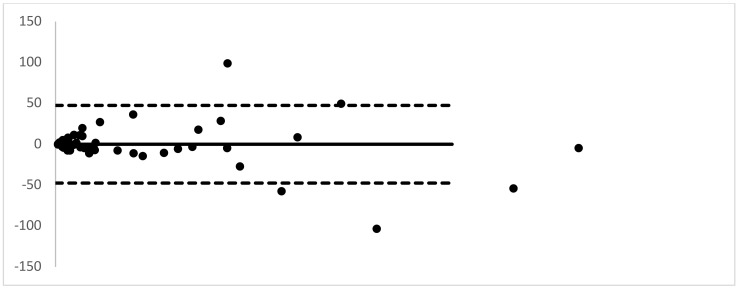
Band Altman Plot to analyse the agreement between manual and autoanalyzer estimated method

**Figure 2:**
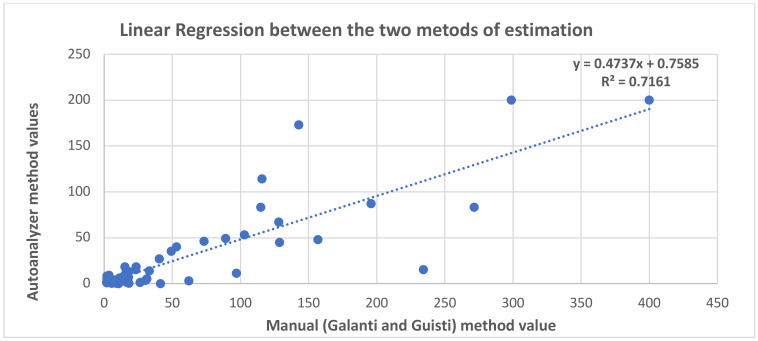
Linear regression between manual and autoanalyzer methods of ADA estimation

Further, the mean difference, bias, Standard Deviation and the upper and lower limits of agreement were determined. The results obtained from the two methods were compared to assess the agreement between the two methods of estimation. A Band Altman plot was generated to estimate the degree of agreement. As shown in Figure 1, 90% of the assayed samples showed agreeable results between the two methods while only a meagre 10% of the samples did not fall within the degree of agreement when analysed by the two methods. Samples showing very high or very low autoanalyzer values tend to show divergence from the expected manual values. Additionally, according to our observation, 71.4% of the divergence was contributed by pleural fluid samples (cases of pleural effusion and multisystem inflammatory syndrome secondary to bronchopneumonia with/without AKI/ hepatitis/ COPD) and 28.5% (focal dyscognitive seizures and neurobrucellosis) of the divergence was accounted for by CSF samples, while none of the serum ADA showed such a divergence between the two assay methods. Further, a linear regression was calculated to predict autoanalyzer (Diazyme assay in cobas 8000) generated ADA values based on the obtained/ observed Galanti and Guisti (manual) method values. A significant regression equation was found (F (1, 57) = 143.58, p < 2.2e-16), with an R2 of 0.711. Study predicted that autoanalyzer ADA levels were found to be equal to 0.7584775 + 0.4737348 (ADA estimated by manual method). The autoanalyser measured ADA value was found to be 0.473 times the value obtained by the Galanti and Guisti (manual) method of ADA estimation (Figure 2).

## Discussion

ADA, one of the key enzymes of purine catabolism involved in the deamination of adenosine, plays a major physiological role in cell mediated immune response by regulating the normal metabolism, proliferation and differentiation of lymphocytes (16). ADA is necessary for proliferation and differentiation of T lymphocytes and monocyte and macrophage and ADA is indispensable requirement for development, differentiation and proliferation of these key components of the immune system cells (12,13) which explains the pathology of SCID. Further, increased serum/fluid ADA is implicated in several diseases involving stimulation of cell mediated immunity such as tuberculosis, pleurisy, tubercular meningitis, typhoid fever, infectious mononeucleosis and bronchogenic carcinoma, etc (16).

Tuberculosis remains a significant public health hazard in India (9,11). Despite the huge advances in the diagnostic techniques for the diagnosis of TB, the challenge of developing a novel, cost-effective, accurate biomarker-based point of care test still prevails. Several diagnostic methods such as tuberculin skin test, interferon-γ release assay, imaging method, pleural biopsy, X-ray, ultrasonography, routine thoracic fluid inspection, the highly sensitive PCR and bacterial culture have been exploited (11,15,17). Rapid definitive diagnosis of TB still remains a challenging area with literature evidences emphasizing the utility of ADA in the diagnosis of TB (12–14). Further, CSF ADA shows a high positive predictive value for ADA test in diagnosis of tuberculous meningitis and ADA has emerged to be a transpicuous and rapid test in the diagnosis of TBM with sensitivity of 0.89, specificity of 0.91 and AUC of 0.96 indicating a sufficient level for diagnostic accuracy (18). With TB and its varied forms being a group of progressive pathologies with severe manifestations and complications and causing considerable mortality and severe neurological morbidity, early diagnosis remains indispensable for rapid treatment and remission and ADA indeed offers an immense potential as an adjunct diagnostic tool for TB and other ADA related pathologies (15,18,19).

Our study showed that though the manual assay technique yielded higher ADA values that the autoanalyzer method, yet introduction of a correction factor of 2.44 could make both the values agreeable. Further, both the methods agreed in their direction of shift (increase or decrease) with PTB and other pulmonary aetiologies showing maximum agreement in elevation of ADA level. The Band Altmann plot showed agreement of the two analytical techniques in almost 90% of the samples. In certain conditions, there was an increase in autoanalyser and manual values, and it was noted that many of these conditions were involved with the respiratory system and both autoanalyser and annual methods were extremely high and proportionate to each other.

However, for a small proportion of samples, the results from two methods were highly disparate and application of the correction factor did not seem to address the observed discrepancy. Though we could not arrive at a definite reason for this discrepancy observed, it is interesting to note that all these samples were derived from patients suffering predominantly from non-infectious conditions and having very low or high ADA values (as obtained from the standard autoanalyzer method). The observed discrepancy could be due to the sample type (serum/CSF/pleural fluid/ascitic fluid), the stage of disease at which the assay was performed, the treatment history or interferences specific to the manual assay technique used. The study is limited by its small sample size and absence of follow-up data. Further, the study did not make any stratified classification of results based on the type of sample (CSF, Ascitic, pleural fluid) analysed and assess the agreement of the two methods specific to each sample type.

The study needs to be replicated with a larger sample size and a stratified model to make definitive conclusions about the alternative manual assay method for ADA. Further, sample type (serum/ CSF/ pleural fluid/ ascitic fluid) specific sensitivity, specificity and limits of detection also need to be investigated in future studies. However, the study definitely shows the huge potential of using an alternative cost effective, feasible manual technique of ADA analysis, the test being inexpensive, relatively non-invasive (compared to invasive conclusive tests like ADA), less time consuming, rapid and reliable. This could make huge difference in analytical and diagnostic approaches in primary healthcare setups of developing countries like India where incidence of PTB and tuberculous meningitis is high.

Thus, we conclude that Galanti and Guisti manual method of ADA estimation might be a feasible, transpicuous and rapid test with high aggregability to the autoanalyzer method and hence reliable for diagnosis of conditions like PTB and TBM. The cost of the cobas 6000/autoanalyzer based diazyme method is higher than the manual method. However, many labs do not have such facilities and hence could resort to the manual method for this investigation.

## References

[1] Weyden MBV, Kelley WN (1976). Human Adenosine Deaminase: distribution and properties. The Journal of Biological Chemistry.

[2] Cristalli G, Costanzi S, Lambertucci C, Lupidi G, Vittori S, Volpini R, Camaioni E (2001). Adenosine deaminase: functional implications and different classes of inhibitors. Medicinal Research Reviews.

[3] AM and, Gennery AR (2018). Adenosine deaminase deficiency: a review. Orphanet J Rare Dis.

[4] Moriwaki Y, Yamamoto T, Higashino K (1999). Enzymes involved in purine metabolism--a review of histochemical localization and functional implications. Histology and Histopathology.

[5] Piras MA, Gakis C, Budroni M, Andreoni G (1978). Adenosine deaminase activity in pleural effusions: an aid to differential diagnosis. Br Med J.

[6] Ocaña I, Martinez-Vazquez JM, Segura RM, Fernandez-De-Sevilla T, Capdevila JA (1983). Adenosine Deaminase in Pleural Fluids: Test for Diagnosis of Tuberculous Pleural Effusion. Chest.

[7] Devkota KC, Shyam BK, Sherpa K, Ghimire P, Sherpa MT, Shrestha R, Gautam S (2012). Significance of adenosine deaminase in diagnosing tuberculous pleural effusion. Nepal Med Coll J.

[8] Sharma SK, Suresh V, Mohan A, Kaur P, Saha P, Kumar A, Pande JN (2001). A prospective study of sensitivity and specificity of adenosine deaminase estimation in the diagnosis of tuberculosis pleural effusion. Indian J Chest Dis Allied Sci.

[9] TBFACTS.ORG (2019). Information about tuberculosis. TB Statistics India.

[10] World Health Organisation (2020). Tuberculosis.

[11] Mallik M, Bhartiya R, Singh R, Kumar M, Bariar NK (2016). Adenosine deaminase: A sensitive and cost-effective method for the detection of tuberculous pleural effusion in a developing state like Bihar, India. Annals of Tropical Medicine and Public Health.

[12] Kothari K, Mistry MA, Goswami YS (2017). Utility of ADA (Adenosine Deaminase) enzyme assay in diagnosis of tuberculous meningitis. Indian Journal of Microbiology Research.

[13] Chikkahonnaiah P, Jaggi S, Goyal B, Garg K, Gupta S, Jaswal S, Kaur K (2017). Utility of serum ADA estimation in the diagnosis of extrapulmonary tuberculosis. Journal of Medical Science and Clinical Research.

[14] Barua R, Hossain MA (2014). Adenosine Deaminase in Diagnosis of Tuberculosis: A Review. Anwer Khan Modern Medical College Journal.

[15] Baba K, Hoosen AA, Langeland N, Dyrhol-Riise AM (2008). Adenosine Deaminase Activity Is a Sensitive Marker for the Diagnosis of Tuberculous Pleuritis in Patients with Very Low CD4 Counts. Plos One.

[16] Varma S, Toppo A (2015). Estimation of serum adenosine deaminase level in patients of pulmonary tuberculosis in a tertiary care hospital in Chhattisgarh. International Journal of Research in Health Sciences.

[17] Gui X, Xiao H (2014). Diagnosis of tuberculosis pleurisy with adenosine deaminase (ADA): a systematic review and meta-analysis. Int J Clin Exp Med.

[18] Pormohammad A, Riahi SM, Nasiri MJ, Fallah F, Aghazadeh M, Doustdar F, Pouriran R (2017). Diagnostic test accuracy of adenosine deaminase for tuberculous meningitis: A systematic review and meta-analysis. Journal of Infection.

[19] Barua R, Hossain MA (2014). Adenosine Deaminase in Diagnosis of Tuberculosis: A Review. Anwer Khan Modern Medical College Journal.

